# RespirAnalyzer: an R package for analyzing data from continuous monitoring of respiratory signals

**DOI:** 10.1093/bioadv/vbae003

**Published:** 2024-01-13

**Authors:** Teng Zhang, Xinzheng Dong, Dandan Wang, Chen Huang, Xiaohua Douglas Zhang

**Affiliations:** Department of Public Health and Medicinal Administration, Faculty of Health Sciences, University of Macau, Taipa, Macau 999078, China; Zhuhai Laboratory of Key Laboratory of Symbolic Computation and Knowledge Engineering of Ministry of Education, Zhuhai College of Science and Technology, Zhuhai 519041, China; Department of Public Health and Medicinal Administration, Faculty of Health Sciences, University of Macau, Taipa, Macau 999078, China; Dr. Neher's Biophysics Laboratory for Innovative Drug Discovery, Macau University of Science and Technology, Taipa, Macau 999078, China; Department of Biostatistics, University of Kentucky, Lexington, KY 40536, United States

## Abstract

**Motivation:**

The analysis of data obtained from continuous monitoring of respiratory signals (CMRS) holds significant importance in improving patient care, optimizing sports performance, and advancing scientific understanding in the field of respiratory health.

**Results:**

The R package *RespirAnalyzer* provides an analytic tool specifically for feature extraction, fractal and complexity analysis for CMRS data. The package covers a wide and comprehensive range of data analysis methods including obtaining inter-breath intervals (IBI) series, plotting time series, obtaining summary statistics of IBI series, conducting power spectral density, multifractal detrended fluctuation analysis (MFDFA) and multiscale sample entropy analysis, fitting the MFDFA results with the extended binomial multifractal model, displaying results using various plots, etc. This package has been developed from our work in directly analyzing CMRS data and is anticipated to assist fellow researchers in computing the related features of their CMRS data, enabling them to delve into the clinical significance inherent in these features.

**Availability and implementation:**

The package for Windows is available from both Comprehensive R Archive Network (CRAN): https://cran.r-project.org/web/packages/RespirAnalyzer/index.html and GitHub: https://github.com/dongxinzheng/RespirAnalyzer.

## 1 Introduction

Respiratory diseases such as apnea syndrome, asthma, and chronic obstructive pulmonary disease (COPD) all pose great threats to the health of patients. Continuous monitoring of respiratory signals (CMRS) is of great significance for the treatment of related diseases (Chen *et al.* 2022). Traditionally, CMRS is mainly achieved through a medical ventilator (Ilia *et al.* 2020). With the development of wearable human respiration measurement systems, the acquisition of respiratory data becomes convenient and fast, which provides people better health services but leads to explosive growth in respiratory data (Yamamoto *et al.* 2019). CMRS data contains a huge amount of physiological and biochemical information that can reflect the health status of the human body. Analyzing and mining the data to find out specific information related to the diseases may generate a new route for medical diagnosis of the diseases (Jin *et al.* 2017, Niu *et al.* 2018).

Fractal and complexity are the internal nonlinear features of respiratory data. Fractality of respiration rate, inter-breath intervals (IBI), and respiration amplitude have been studied (Peng *et al.* 2002, Fadel *et al.* 2004). Fractal analysis methods, such as Lyapunov exponents, detrended fluctuation analysis (DFA) and power spectral density (PSD) have been applied for the analysis of respiratory data (Fadel *et al.* 2004, Kapidzic *et al.* 2014). Multiscale sample entropy (MSE) of respiratory data has been used to examine the complexity in the cardiopulmonary system (Veiga *et al.* 2010, Veiga *et al.* 2011, Chen *et al.* 2017, Platiša *et al.* 2020). The R packages have been important tools for extracting fractal and complexity features in data from continuous monitoring of physiological signals including respiratory signal. Although the R packages “nonlinearTseries,” “MSMVSampEn,” and “CGManalyzer” can calculate sample entropy and/or MSE (Chen *et al.* 2019a) and the packages *DFA* and *MFDFA* can conduct fractal analysis, there is still a lack of comprehensive analytic tools specifically for feature extraction, fractal and complexity analysis of CMRS data. Therefore, we developed this R package to fill this gap, which is important with the rapid accumulation of CMRS data.

## 2 Methods

### 2.1 Data for the demonstration of *RespirAnalyzer*

We utilized respiratory data obtained from the Fantasia database, available on PhysioNet (https://physionet.org/content/fantasia/1.0.0/), to illustrate the application of *RespirAnalyzer* in the analysis of CMRS data. The dataset comprises 20 individuals in good health within two age groups: 21–34 years for the young cohort and 68–85 years for the elderly cohort. Throughout the measurements, all subjects maintained a supine position, engaged in rest, and watched the movie “Fantasia” to ensure wakefulness.

The respiratory data were originally recorded at a sampling frequency of 250 Hz and spanned a duration of 120 min. To enhance computational efficiency, we resampled the respiratory data to a frequency of 50 Hz during the download from PhysioNet. Subsequent to data acquisition, we identified the presence of false signals and noise. Consequently, we excluded two datasets (one from each age group) characterized by excessive false signals and noise, resulting in a final dataset of 19 sets for both the young and elderly individuals.

### 2.2 Methods for complexity and fractality

*RespirAnalyzer* offers the capability to compute MSE, multifractal detrended fluctuation analysis (MFDFA), and PSD. The MSE algorithm, as devised by Costa *et al.* (2002), involves two integral steps: (i) calculating sample entropy and (ii) using a coarse-graining procedure to generate a set of time series equations depicting system dynamics across various time scales.

MFDFA, an extension of DFA, was initially introduced by Kantelhardt *et al.* (2002). Subsequently, they proposed a modified version suitable for negative generalized Hurst exponents in 2009 (Kantelhardt 2009). While originally designed for multifractal analysis of time series, MFDFA exhibits versatility by application to higher dimensions, as demonstrated by Gu and Zhou (2006) and Zhou *et al.* (2013).

To model multifractal behaviors, an extended binomial multifractal model has been developed. This model, reliant on two parameters α and β, is instrumental in fitting MFDFA results to real data (Kantelhardt *et al.* 2006). Based on this model, the generalized Hurst exponent is.

(1) $$h\left(q\right)=\frac{1}{q}-\frac{\ln\left(\alpha^{q}\;+\;\beta^{q}\right)}{q\ln\left(2\right)}$$

Based on this relationship, our function *fit.model* uses nonlinear regression to fit the generalized Hurst exponent obtained from MFDFA analysis of IBI series. The output of *fit.model* is the estimated values of α, β and the goodness of fit (i.e. Pearson correlation coefficient between observed Hurst exponent and its predicted value from the extended BMF model). Based on the estimated values of α and β, parameters $h_{\min}$, $h_{\max}$ and Δh can be calculated

(2) $$h_{\min}=\underset{q\to +\infty }{\lim}h\left(q\right)=-\frac{\ln\alpha }{\ln\left(2\right)}$$

(3) $$h_{\max}=\underset{q\to -\infty }{\lim}h\left(q\right)=-\frac{\ln\beta }{\ln\left(2\right)}$$

(4) $$\Delta h=h_{\max}-h_{\min}=\frac{\ln\alpha -\ln\beta }{\ln\left(2\right)}$$

where $h_{\min}$ and $h_{\max}$ represent the scaling behaviors of the weakest and strongest fluctuations respectively, and Δh represents the degree of multifractal. For a large Δh, the multifractal fluctuations are strong; for Δh≈0, the multifractal is almost vanishing.

*RespirAnalyzer* uses the ^low^PSD algorithm, as introduced by Eke *et al.* (2000), to accurately compute the PSD of biomedical time series data.

The detailed algorithms to calculate MSE, MFDFA, the extended binomial multifractal model and PSD are described in the Supplementary Materials.

## 3 Results

*RespirAnalyzer* has been developed specifically for the comprehensive analysis of CMRS data, emphasizing complexity and fractality assessments. Table 1 provides a succinct comparative overview of *RespirAnalyzer* alongside existing software tools dedicated to the analysis of complexity and fractality. Further software details are provided in the Supplementary Materials.

**Table 1.** Comparison of analysis on complexity and fractality in multiple major existing packages.^a^

Package name	Core function for complexity and fractality	Key feature	Focused data
*RespirAnalyzer*	*MSE, LowPSD, MFDFA*,	Multiscale entropy, PSD, multifractal detrended fluctuation	CMRS data
*CGManalyzer*	*MSEbyC.fn*	Multiscale sample entropy	CGM data
*nonlinearTseries*	*sampleEntropy*	Sample entropy	1D time series
*MSMVampEn*	*MSMVSampEn*	Multiscale multivariate sample entropy	High-dimensional time series
*psd*	*pspectrum*	PSD	1D time series
*MFDFA*	*MFDFA*	Multifractal detrended fluctuation	1D time series

a *RespirAnalyzer* has main functions *find.peaks, MovingAverage, MSE, LowPSD, MFDFA, fit.model, MFDFAplot.fn, Seriesplot.fn, Individualplot.fn, GroupComparison.fn, Groupplot.fn.* The detailed description of these functions can be found in https://cran.r-project.org/web/packages/RespirAnalyzer/index.html. In addition, a corresponding development repository is made available at https://github.com/dongxinzheng/RespirAnalyzer. For demonstration, we used respiratory data sourced from the Fantasia database to elucidate the procedure of utilizing RespirAnalyzer for the analysis of CMRS data. The corresponding code is included in the Supplementary Materials for reference. The primary functionalities are described as follows.

### 3.1 Identification of peaks and calculation of IBI

**Figure 1.**
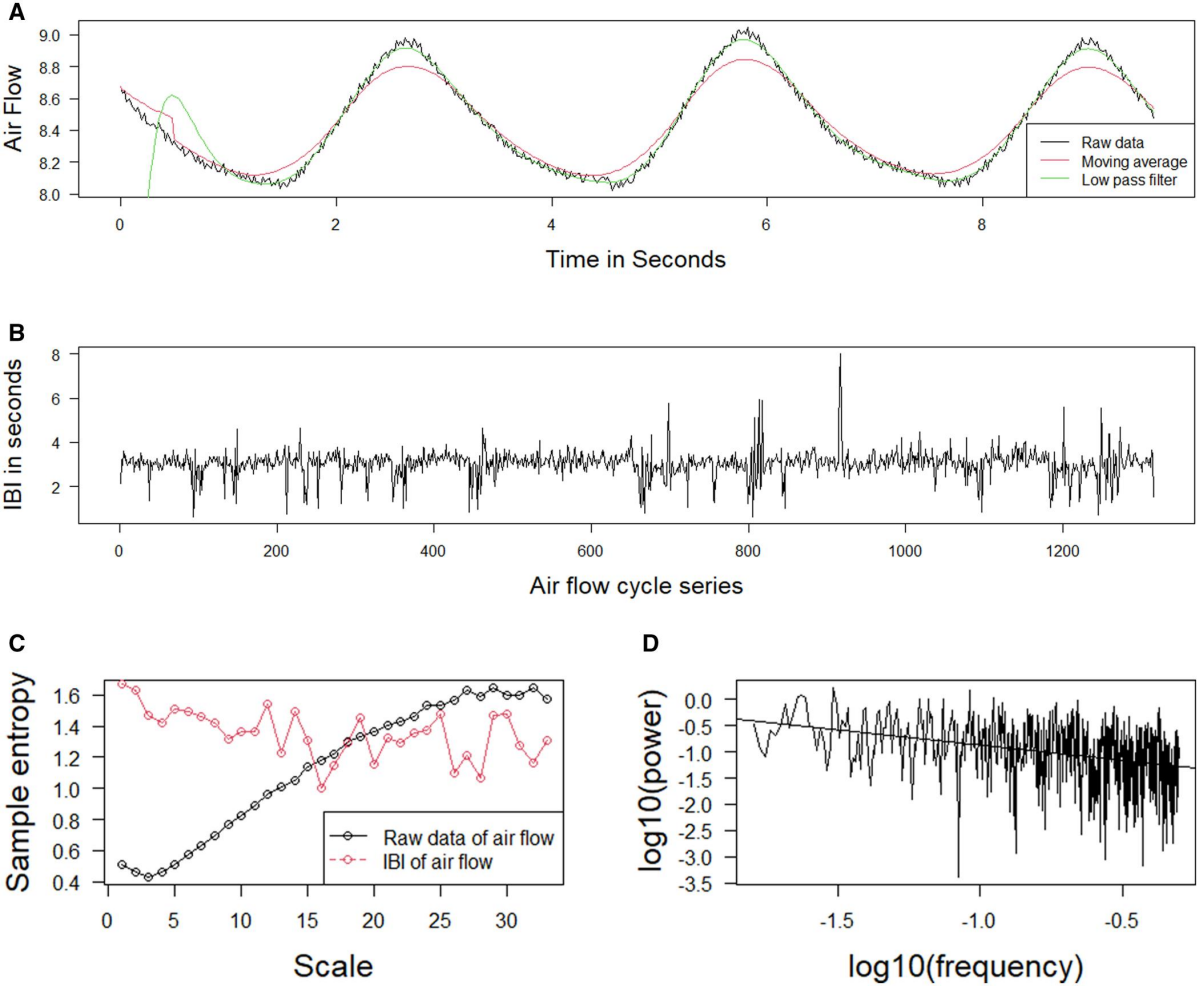
Illustrating the use of R functions for MSE and PSD analysis. The function *fit.model* displays the air flow data from a single young individual within the Fantasia database (A); *find.peaks* derives and shows Inter-Breath Intervals (IBI) series (B); *MSE* computes and displays multi-scale sample entropy of raw and IBI of air flow (C); *lowPSD* conduct PSD analysis on IBI of air flow (D).

CMRS data have a rather unique feature, namely one breathing waveform after another, as shown in Fig. 1A obtained using the function *Seriesplot.fn*. Because of breathing cycle existing in CMRS data, it is critical to identify the time and value of each peak in a cycle and then to derive IBI. The function *find.peaks* can identify the time and height of a peak in a cycle of CMRS. To reduce the impact of random noise as shown in the black line of Fig. 1A, two approaches (i.e. moving average and low pass filter) are implemented in *find.peaks*. Figure 1A also shows the results of finding peaks using moving average (red line) and low pass filer (green line). The IBI for a cycle is then the difference of time of two adjacent peaks. The IBI series for all the data is shown in Fig. 1B.

### 3.2 MSE for complexity analysis

The MSE analysis can be used to investigate the complexity of CMRS data either in original scale or derived IBI format, as shown in Fig. 1C. RespirAnalyzer provides the function *MSE* for streamlined MSE analysis, akin to the function *MSEbyC.fn* in the package CGManalyzer (Zhang *et al.* 2018). The syntax is *MSE(x, tau, m, r, I)*. To enhance user experience, we have conducted additional packaging work in the function *MSE*. This includes optimizing the functionality for calling its corresponding C source file in RespirAnalyzer, making the utilization of *MSE* more straightforward and user-friendly than *MSEbyC.fn* for calculating sample entropy. The C programs called by *MSE* and *MSEbyC.fn* have undergone adaptations, presenting an evolved iteration of mse.c sourced from http://www.physionet.org/ (Costa *et al.* 2002). This enhanced version is designed to handle substantially longer data series, surpassing the capabilities of its predecessor.

### 3.3 PSD for fractal analysis in frequency domain

Previous studies have proven that fractal nature exists in the IBI series (Peng *et al.* 2002, Fadel *et al.* 2004), not in original respiratory data. Therefore, PSD is applied to the IBI data. In our package, the function *LowPSD* can be used to conduct fractal analysis on the IBI series in the frequency domain and draw a plot of log power versus log frequency (Fig. 1D). The linear relationship between log power and log frequency proves the fractality of IBI series and the slope of the linear regression line is the measure of fractality in frequency domain (−β = 1.443 in Fig. 1D).

### 3.4 MFDFA for fractal analysis in time domain

**Figure 2.**
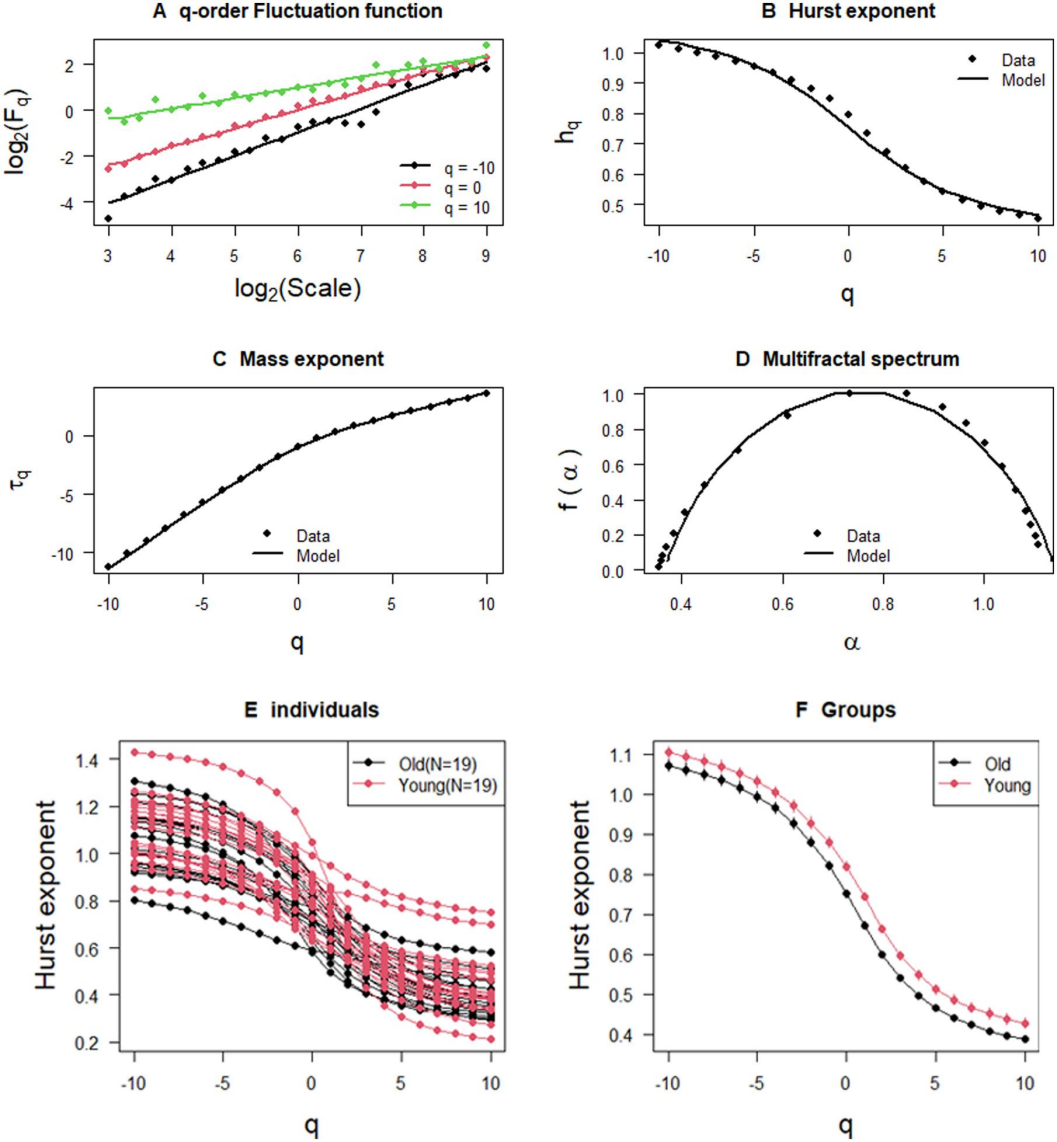
MFDFA analysis using R functions *MFDFAplot.fn* for a single young individual (A–C) and *Individualplot.fn* and *Groupplot.fn* for comparing IBI series of 19 old and 19 young individuals from the Fantasia database (E, F). (A) q-order fluctuation functions showing the existence of multifractal in the IBI series, (B) Hurst exponent (i.e. *hq*) indicating a persistent IBI series, (C) mass exponent (i.e. $\tau_{q}$), (D) multi-fractal spectrum showing the distribution of scaling exponents for a signal, (E) Hurst exponents for each individual subject, (F) mean and standard error of Hurst exponent by groups of subjects which shows the differentiation of Hurst exponent between old and young people.

While the function *PSD* can explore the fractality in frequency domain, the function *MFDFA* can investigate the fractality in time domain. We independently developed a C++ program MFDFA.cpp that is included in this package to enhance the processing speed of large datasets, drawing inspiration from the implementation in the R package MFDFA (Kantelhardt *et al.* 2002, Laib 2018), and then wrapped it in the R function *MFDFA*. Our MFDFA.cpp is also available at https://github.com/dongxinzheng/RespirAnalyzer/blob/main/src/MFDFA.cpp. The *MFDFA* can be used to calculate q-order fluctuation function, Hurst exponent, mass exponent and multifractal spectrum for an individual subject. These results computed from *MFDFA* can be displayed using another function *MFDFAplot.fn* in our package. Figure 2A–D displays q-order fluctuation function, Hurst exponent, mass exponent and multifractal spectrum, respectively, on the IBI data of an individual subject.

### 3.5 Modeling MFDFA for fractal analysis

Our function *MFDFAplot.fn* can display the fitted values from the extended BMF model, as shown in solid black curves in Fig. 2B–D. These curves show that the model fits the MFDFA results very well.

### 3.6 Group comparison

It is a common task to compare complexity and fractality in different types of subjects. Our functions *Individualplot.fn* and *Groupplot.fn* can be used to display the calculated results of complexity and fractality. *Individualplot.fn* can plot the results of individual subjects one by one with labeling the type that an individual subject belongs to (Fig. 2E) whereas *Groupplot.fn* can plot the mean and error bar of the results by the types of subjects (Fig. 2F). The error bar can be a standard deviation or standard error which can be calculated using the function *GroupComparison.fn*. *GroupComparison.fn* can calculate the number of samples, mean, standard deviation, standard error, median, confident interval and *P*-value of ANOVA for each type of subjects. Supplementary Table S1 and Fig. 2F show analytic results on the differences of Hurst exponent between old and young groups in the example data, which reveals a significant disparity in Hurst exponent at *q* = 0,1,2 between old and young individuals.

## 4 Discussion

The main task for preprocessing CMRS data is to identify the peak or trough of the data. After identifying the peaks, we can calculate IBI, respiratory intensity and respiratory rate which are important physiological parameters of the respiratory system (Ni *et al.* 2021). In addition, the fractality of CMRS data has been proven to be contained in IBI series which represent the peak-to-peak intervals of CMRS data (Peng *et al.* 2002, Fadel *et al.* 2004). Therefore, finding peaks is the first step for analyzing CMRS data. Because measurement of CMRS data is susceptible to interference, the collected data is usually contaminated by noise, leading to fake peaks in the raw data. Low pass filter and moving average are two methods to reduce the impact of noise through smoothing local trends (Peng *et al.* 2002). Our function *find.peaks* allows users to choose one of these two methods so that true peaks of CMRS data can be identified.

Our package implements three major methods (*MSE*, *LowPSD*, *MFDFA*) and one model (*fit.model*) for fractal and complexity analysis of CMRS data. Entropy can quantify the complexity of data and many entropy measures have been developed and applied for the analysis of respiratory data (Jin *et al.* 2017). Among them, MSE analysis can measure the overall complexity of the original time series by calculating the sample entropy of the coarse-grained time series (Costa *et al.* 2005) and have been applied for the research on a variety of diseases (Costa *et al.* 2005, Thomas *et al.* 2015, Chen *et al.* 2017, 2019a,b, Jin *et al.* 2017, Zhang *et al.* 2017). Multiple R packages are available for calculating sample entropy, and a comparative analysis on them can be found in a published article (Chen *et al.* 2019a). The function *MSE* in *RespirAnalyzer* offers a distinct advantage over the function *MSEbyC.fn* in CGManalyzer for calculating sample entropy. This advantage lies in its enhanced user-friendliness, thanks to additional packaging efforts.

The function *lowPSD* implements a reliable algorithm (Eke *et al.* 2000) to calculate the PSD of biomedical time series to conduct fractal analysis of time series in the frequency domain. Mandelbrot first proposal the definition of fractal geometry and developed it into a system theory in 1975 (Mandelbrot 1975). The fractality in frequency domain exists when the time series shows the power-law relationship on the frequency scale. Peng *et al.* (1994) developed DFA. Kandelhardt *et al.* (2002) extend the DFA to study multifractal processes, giving rise to MFDFA. With the application of DFA, the fractality of the IBI series in time domain has been studied (Peng *et al.* 2002, Fadel *et al.* 2004), but researches on the multifractality are limited. The MFDFA is a potential method for measuring the multifractal of IBI series, which has not been applied for the analysis of respiratory data yet although it has been applied in heart rate (Ivanov *et al.* 1999, Nunes Amaral *et al.* 2001) and blood glucose (Thomas *et al.* 2015, Chen *et al.* 2019b). Researchers have used the extended BMF model to fit the multifractal analysis results of real data successfully, such as the river runoff record and solar wind record (Kantelhardt *et al.* 2006, Macek 2006). The function *fit.model* uses a nonlinear regression method to fit the results obtained from MFDFA analysis of IBI series.

In short, we developed a comprehensive set of analytic tools specifically tailored for feature extraction and the fractal and complexity analysis of CMRS data. The features and applicability of several major existing R packages capable of implementing sample entropy, PSD, and MFDFA calculations is outlined and discussed in the supplementary material.

The nonlinear features, specifically PSD, MFDFA, and MSE, hold significant clinical relevance for advancing research in the field of respiratory diseases. For example, PSD serves as a tool to unveil the fractal characteristics of a data series in the frequency domain. Previous investigations have demonstrated the presence of fractal nature in CMRS data, with studies revealing alterations in the fractal scaling properties of human respiratory dynamics linked to factors such as healthy aging and gender (Peng *et al.* 2002, Fadel *et al.* 2004). MFDFA extends the exploration of fractal characteristics, focusing on the time domain. While the fractal nature of CMRS data in the time domain has been examined using DFA, MFDFA has not been previously applied to the analysis of airflow data. MFDFA holds promise as a method capable of measuring the multifractality of CMRS data, as evidenced by its diverse applications in heart rate (Nunes Amaral *et al.* 2001) and blood glucose (Thomas *et al.* 2015). The observation of a significant disparity in Hurst exponent at *q* = 0,1,2 between old and young individuals shown in Fig. 1F and Supplementary Table S1 implies that the multifractal characteristics of airflow data undergo changes with age, though the clinical mechanisms behind this phenomenon remain poorly understood. Future studies can aim to delve deeper into whether such changes also manifest in patients with respiratory diseases. We aspire to leverage multifractal features for the diagnosis and early detection of respiratory ailments. MSE is used to assess the complexity within the cardiopulmonary system (Veiga *et al.* 2010). For instance, previous studies have shown that the sample entropy of respiratory data in young females is lower than that in young males (Kapidzic *et al.* 2014), and specific cardiac rhythms in heart failure patients can induce alterations in MSE of respiratory data (Platiša *et al.* 2020).

In *RespirAnalyzer*, we used a comprehensive approach exploring three nonlinear features (i.e. PSD, MFDFA, and MSE) of CMRS data. These features have clinical application for research in human respiratory system. As a promising avenue for future research, our focus will extend to investigating the clinical applications and implications of the nonlinear features computed by *RespirAnalyzer*. This exploration aims to assess the diagnostic potential and treatment evaluation utility of these features in the context of respiratory diseases, such as asthma and COPD.

Given that the fractal features of the airflow data manifest in the IBI sequence, conducting airflow data analysis necessitates the extraction of peaks. Raw airflow data is particularly susceptible to noise. To mitigate this, the function *find.peak* incorporates algorithms such as moving average and low-pass filters, strategically used to diminish the impact of noise during peak extraction. Additionally, it is crucial to consider the sampling rate of the airflow data. Therefore, further investigation into the interplay of noise and sampling rates in peak extraction is warranted.

## Supplementary Material

vbae003_Supplementary_Data
