# Supplementary material for: RespirAnalyzer: an R package for analyzing data from continuous monitoring of respiratory signals
Source: Bioinform Adv. 2024 Jan 13;4(1):vbae003. doi: 10.1093/bioadv/vbae003 (PMC10807906; doi:10.1093/bioadv/vbae003)
Supplement: vbae003_Supplementary_Data [file vbae003_supplementary_data.docx]

Supplementary Materials

RespirAnalyzer: an R package for analyzing data from continuous monitoring of respiratory signals

Teng Zhang^1,#^, Xinzheng Dong^2,#^, Dandan Wang^1^, Chen Huang^3^, Xiaohua Douglas Zhang^4,*^

^1^Faculty of Health Sciences, University of Macau, Taipa, Macau.

^2^Zhuhai Laboratory of Key Laboratory of Symbolic Computation and Knowledge Engineering of Ministry of Education, Zhuhai College of Science and Technology, Zhuhai 519041, China

^3^Dr. Neher's Biophysics Laboratory for Innovative Drug Discovery, Macau University of Science and Technology, Taipa, Macau

^4^Department of Biostatistics, University of Kentucky, Lexington, KY 40536, USA

1. **Methods for complexity and fractality**
   1. **Multiscale entropy**

The MSE algorithm is composed of two steps [1]:

(1) ***The algorithm for calculating the sample entropy***. For the time series $\{x_{1},\ldots,x_{i},\ldots,x_{N}\}$ with the length of *N*. A new vector could be defined by $X_{i}^{m}=\{x_{i},x_{i+1}...,x_{i+m-1}\}$ and *m* is dimension (length of the vector). The distance between vectors $X_{i}^{m}$ and $X_{j}^{m}$ is defined as maximum absolute difference between corresponding elements:

$$\begin{aligned} d\left( X_{i}^{m},X_{j}^{m} \right)=\max_{k=1,2,\ldots,m} \left| u\left( i+k-1 \right)-u\left( j+k-1 \right) \right|\#\left( 1 \right) \end{aligned}$$

The $X_{i}^{m}$ and $X_{j}^{m}$ is a matched vector pair when $d\left[ X_{i}^{m},X_{j}^{m} \right]\leq r,j=1,2,\ldots,N-m,i\neq j$, where the *r* is a pre-defined threshold. Let $n_{i}^{m}(r)$ represent the number of matched vector pair and $P_{i}^{m}\left( r \right)$ represent the relative frequency of matched vector pair.

$$\begin{aligned} P_{i}^{m}\left( r \right)=\frac{n_{i}^{m}\left( r \right)}{N-m-1}\#\left( 2 \right) \end{aligned}$$

Apply the same operation for the dimension of $m+1$ to get $P_{i}^{m+1}\left( r \right)$. $P^{m}\left( r \right)$ and $P^{m+1}(r)$ are the mean of $P_{i}^{m}(r)$ and $P_{i}^{m+1}\left( r \right)$.

$$\begin{aligned} P_{i}^{m+1}\left( r \right)=\frac{n_{i}^{m+1}\left( r \right)}{N-m-1}\#\left( 3 \right) \end{aligned}$$

$$\begin{aligned} P^{m}\left（ r \right）=\frac{1}{N-m}\sum_{i=1}^{N-m} P_{i}^{m}\left( r \right)\#\left( 4 \right) \end{aligned}$$

$$\begin{aligned} P^{m+1}\left( r \right)=\frac{1}{N-m}\sum_{i=1}^{N-m} P_{i}^{m+1}\left( r \right)\#\left( 5 \right) \end{aligned}$$

The sample entropy of the time series could be expressed as follow:

$$\begin{aligned} SampEn\left( m,r \right)=-\lim_{N\to\infty} log\frac{P^{m+1}\left( r \right)}{P^{m}\left( r \right)}\#\left( 6 \right) \end{aligned}$$

(2) ***A coarse-graining procedure to derive a set of time series equations representing the system dynamics on different time scales***. For a univariate discrete signal $\{x_{1},\ldots,x_{i},\ldots,x_{N}\}$ with the length of *N*, the coarse-grained time series $\left\{ y^{\tau} \right\}$ for scale *τ* is obtained by averaging samples of the time series inside a consecutive but non-overlapping window of length *τ*.

$$\begin{aligned} y_{j}^{\tau}=\frac{1}{\tau}\sum_{i=\left( j-1 \right)\tau+1}^{j\tau} x_{i},1\leq j\leq\frac{N}{\tau}\#\left( 7 \right) \end{aligned}$$

When scale τ =1, the coarse-grained time series $\left\{ y^{\tau} \right\}$ corresponds to the original signal. As the change of scale τ, we construct different coarse-grained time series and subsequently calculate sample entropy of the newly coarse-grained time series $\left\{ y^{\tau} \right\}$. The sample entropy on different scales is MSE.

- 1. **Multifractal detrended fluctuation analysis (MFDFA)**

MFDFA is a method developed from detrended fluctuation analysis (DFA) for characterizing multifractal behaviors of unsteady time series and noise. The method was first proposed by Kantelhardt et al. [3], and then they proposed a modified method to make it suitable for negative generalized Hurst exponent [4]. Although the method is designed for multifractal analysis of time series, it can also be applied to higher dimensions [5, 6]. The algorithm for MFDFA analysis is as follows.

(1) Let $X\left( t \right)=\{X\left( t_{1} \right), X\left( t_{2} \right),\ldots,X\left( t_{N} \right)\}$ denote a biomedical time series with a length of N. The first step is to subtract the mean $\bar{X}$ to get a new cumulative sequence:

| $Y\left( i \right)=\sum_{k=1}^{i} X(k)-\bar{X} i=1,\ldots,N$ | (8) |
| --- | --- |

Where $\bar{X}$ is the mean of a biomedical time series and $Y\left( i \right)$ is named as a cumulative profile.

(2) The profile $Y\left( i \right)$is divided into $N_{s}$ small nonoverlapping segments and the number of points in each small segment is *s*, where $N_{s}=int(\frac{N}{s})$. The length *N* of the time series may not be divisible by scale *s* and the remaining data may not be used. To make full use of the length of the time series, the time series can be again divided into $N_{s}$ small segments from the opposite direction of the data by the same scale *s*, so that a total of ${2N}_{s}$ segments can be obtained.

(3) In each segment, the data is fitted using the least squares method to obtain the fitting polynomial $y_{v}(i)$, and then the root mean square deviation of each segment is given by the following:

| $F^{2}\left( v,s \right)=\frac{1}{s}\sum_{i=1}^{s} \left\{ Y\left[ \left( v-1 \right)s+i \right]-y_{v}\left( i \right) \right\}^{2},v=1,\ldots,N_{s}$ | (9) |
| --- | --- |
| $F^{2}\left( v,s \right)=\frac{1}{s}\sum_{i=1}^{s} \left\{ Y\left[ N-\left( v-1 \right)s+i \right]-y_{v}\left( i \right) \right\}^{2},v=N_{s}+1,\ldots,2N_{s}$ | (10) |

In this step, the order of the fitting polynomial $y_{v}(i)$ is $v$, which is a positive real number. Since the detrending of the time series is done by the subtraction of the polynomial fits from the profile, different order $v$ has different capabilities in eliminating series trends.

(4) Average over all the 2$N_{s}$ segments and obtain the fluctuation function $F(s)$.

| $F\left( s \right)=\left[ \frac{1}{2N_{s}}\sum_{v=1}^{2N_{s}} F^{2}\left( v,s \right) \right]^{1/q}$ | (11) |
| --- | --- |

(5) If the biomedical time series $X\left( t \right)=\{X\left( t_{1} \right), X\left( t_{2} \right),\ldots,X\left( t_{N} \right)\}$ is self-similarity, the power law relationship is satisfied between the q-oder fluctuation function and the time scale$s$ :

| $F_{q}\left( s \right)\propto\delta s^{h\left( q \right)}$ | (12) |
| --- | --- |

where $\delta$ is a constant and function $h(q)$ is generalized Hurst exponent which is depend on *q* for multifractal time series.

- 1. **The extended** **binomial multifractal (BMF) model**

An extended BMF model has been proposed, which uses two parameters $\alpha$ and $\beta$ [7]:

| $x_{k}=\alpha^{n\left( k-1 \right)}\beta^{n_{max}-n\left( k-1 \right)};0.5<\alpha<\beta<1$ | (13) |
| --- | --- |

Thus, the multifractal result of the extended BMF model will change.

| $p_{2s}\left( v \right)=p_{s}\left( 2v-1 \right)+p_{s}\left( 2v \right)=\left[ \frac{\beta}{\alpha}+1 \right]p_{s}\left( 2v \right)$ | (14) |
| --- | --- |

The partition function of the model is

| $Z_{q}\left( s \right) = \sum_{v=1}^{N/s} \left[ p_{s}\left( v \right) \right]^{q} = \sum_{v=1}^{N/2s} {\{[p_{s}\left( 2v-1 \right)]}^{q}+\left[ p_{s}\left( 2v \right) \right]^{q}\}$ $=\left( \frac{\beta^{q}}{\alpha^{q}}+1 \right)\sum_{v=1}^{N/2s} \left[ p_{s}\left( 2v \right) \right]^{q}=\frac{\alpha^{q}+\beta^{q}}{\left( \alpha+\beta\right)^{q}}\sum_{v=1}^{N/2s} \left[ p_{2s}\left( v \right) \right]^{q}$ $\begin{aligned} =\frac{\alpha^{q}+\beta^{q}}{\left( \alpha+\beta\right)^{q}}Z_{q}\left( 2s \right)\# \end{aligned}$ | (15) |
| --- | --- |

The mass exponent and the generalized Hurst exponent are

| $\tau_{q}=\tau\left( q \right)=\frac{q\ln\left( \alpha+\beta\right)-\ln\left( \alpha^{q}+\beta^{q} \right)}{\ln\left( 2 \right)}$ | (16) |
| --- | --- |
| $h_{q}=h\left( q \right)=\frac{1}{q}-\frac{\ln\left( \alpha^{q}+\beta^{q} \right)}{q\ln\left( 2 \right)}+\frac{\ln\left( \alpha+\beta\right)}{\ln\left( 2 \right)}$ | (17) |

It is easy to know that $h\left( 1 \right)=1$ for all values of $\alpha$ and $\beta$, so this model can only fit the multifractal results that satisfied $h\left( 1 \right)=1$. This will greatly limit the application of this model. Therefore, we plan to subtract the offsets $\frac{ln(\alpha+\beta)}{ln(2)}$ so that the$h\left( 1 \right)$ can be any possible values. The new expression of generalized Hurst exponent is

| $h\left( q \right)=\frac{1}{q}-\frac{\ln\left( \alpha^{q}+\beta^{q} \right)}{q\ln\left( 2 \right)}$ | (18) |
| --- | --- |

Based on the expression of the generalized Hurst exponent, we can also find that the limit values of $h\left( q \right)$ and they are equal to

| $h_{min}=\lim_{q\to+\infty} h\left( q \right)=-\frac{\ln\alpha}{\ln\left( 2 \right)}$ | (19) |
| --- | --- |
| $h_{max}=\lim_{q\to-\infty} h\left( q \right)=-\frac{\ln\beta}{\ln\left( 2 \right)}$ | (20) |

In addition, two new parameters can be calculated as follows.

| $\Delta h=h_{max}-h_{min}=\frac{\ln\alpha-\ln\beta}{\ln\left( 2 \right)}$ | (21) |
| --- | --- |
| $R=\frac{h_{max}-h\left( 0 \right)}{h\left( 0 \right)-h_{min}}=1$ | (22) |

In theory, all the parameters $h_{min}, h_{max}, \Delta h, R$ are meaningful because they can measure multifractal behaviors. The $h_{min}$ describe the scaling behaviors of the largest fluctuation; the $h_{max}$ describe the scaling behaviors of weakest fluctuation; $\Delta h$ corresponds to the degree of multifractal; and the $R$ reflects the symmetrical of multifractal distribution. Eq. (22) proves that $R$ is equal to 1 for the extended binomial multifractal model, which means that the multifractal distribution of extended BMM is symmetrical. With the introduction of these new parameters, we can more comprehensively describe the multifractal behaviors of biomedical time series.

- 1. **Power spectral density (PSD)**

In the package, the function “lowPSD” applies the most reliable algorithm ^low^PSD to calculate PSD of biomedical time series [2]. The specific calculation steps are as follows.

1. Subtract the mean of the time series from the time series to obtain a new mean-removed time series;
2. Perform Fast Fourier Transform on the new mean-removed time series to obtain the power spectrum function $P(f)$;
3. Draw the log-log plot of the power spectrum function, and the high-frequency power estimates need to be excluded. The $\beta$ is the slope of the fitting line.
4. **Group comparison of complexity and fractality features in the example**

We use RespirAnalyzer to investigate the features of complexity and fractality described in Section 2 to the data of example described in Section 1. The results are shown in Figures 1 and 2. The comparison of Hurst exponent between old and young people is shown in Table S1, which indicates that Hurst exponent at q=0,1, 2, …, has statistically significant difference between old and young people.

Table S1. Group comparison of Hurst exponent between old and young people

| Variables | Old | Young | P value |
| --- | --- | --- | --- |
| Number | 19 | 19 | - |
| Age | 68-81 | 21-34 | - |
| Hurst exponent (q=-10) | 1.07±0.14 | 1.10±0.14 | 0.471 |
| Hurst exponent (q=-9) | 1.06±0.14 | 1.09±0.14 | 0.464 |
| Hurst exponent (q=-8) | 1.05±0.14 | 1.08±0.14 | 0.455 |
| Hurst exponent (q=-7) | 1.03±0.14 | 1.07±0.14 | 0.444 |
| Hurst exponent (q=-6) | 1.02±0.14 | 1.05±0.14 | 0.428 |
| Hurst exponent (q=-5) | 0.99±0.14 | 1.03±0.14 | 0.406 |
| Hurst exponent (q=-4) | 0.96±0.13 | 1.00±0.14 | 0.374 |
| Hurst exponent (q=-3) | 0.93±0.13 | 0.97±0.14 | 0.328 |
| Hurst exponent (q=-2) | 0.88±0.12 | 0.93±0.14 | 0.257 |
| Hurst exponent (q=-1) | 0.82±0.11 | 0.88±0.13 | 0.153 |
| **Hurst exponent (q=0)** | **0.75±0.10** | **0.82±0.12** | **0.048** |
| **Hurst exponent (q=1)** | **0.67±0.09** | **0.75±0.10** | **0.022** |
| **Hurst exponent (q=2)** | **0.60±0.08** | **0.66±0.09** | **0.027** |
| Hurst exponent (q=3) | 0.54±0.08 | 0.60±0.10 | 0.06 |
| Hurst exponent (q=4) | 0.50±0.08 | 0.55±0.11 | 0.107 |
| Hurst exponent (q=5) | 0.47±0.07 | 0.51±0.12 | 0.148 |
| Hurst exponent (q=6) | 0.44±0.07 | 0.49±0.12 | 0.18 |
| Hurst exponent (q=7) | 0.42±0.07 | 0.47±0.13 | 0.204 |
| Hurst exponent (q=8) | 0.41±0.08 | 0.45±0.13 | 0.222 |
| Hurst exponent (q=9) | 0.40±0.08 | 0.44±0.13 | 0.235 |
| Hurst exponent (q=10) | 0.39±0.08 | 0.43±0.13 | 0.246 |

1. **Discussion of existing software for the analysis of complexity and fractality**

In RespirAnalyzer package, we used three methods to analyze complexity and fractality in data derived from CMRS, namely power spectral density, multifractal detrended fluctuation analysis and multiscale sample entropy.

The comparison of the key features and applicability of RespirAnalyzer with multiple existing packages for analyzing complexity and fractality is outlined in Table 1 and discussed as follows.

*CGManalyzer,* an R package for analyzing continuous glucose monitoring studies. The package contains function *MSEbyC.fn* to call a C function to calculate MSE of an equally spaced time series. The function *MSE* in RespirAnalyzer is more user-friendliness to *MSEbyC.fn* because of additional packaging efforts.

*nonlinearTseries*, a package focuses on nonlinear analysis of time series. The function sampleEntropy calculates sample entropy. Before calculating entropy with this package, correlation dimension should be precomputed, then sample entropy can be generated by dividing correlation sums in different embedding dimensions.

*psd,* a package for adaptive power spectral density estimation using optimal sinusoidal multitaskers. The package contains function *pspectrum,* which returns power spectral density estimates of a timeseries, with an optimal number of tapers at each frequency based on iterative reweighted spectral derivatives.

*MSMVampEn*, the computing program is designed to compute MMSE. Under particular parameter settings, it can calculate multivariate sample entropy, MSE, or multivariate MSE.

*MFDAF*, a package for multifractal detrended fluctuation analysis of a time series. The package contains function *MFDFA* to calculate MFDFA of a time series.

1. **Codes for demonstrating the use of *RespirAnalyzer***

if( !require("RespirAnalyzer") ) install.packages( "RespirAnalyzer" )

library(RespirAnalyzer)

# load Data from TestData dataset

data("TestData")

########################### Figure 1 ############################

par( mfrow=c(2,1), mar=c(5.1, 5.1, 3.1, 2.1) )

##### Figure 1A: Raw data of air flow

# Moving Average

W <- Fs <- 50

fit.MA <- MovingAverage(Data[,2],W)

# Low pass filter

bf <- signal::butter(2, 2/Fs, type="low")

fit.LPF <- signal::filtfilt(bf, Data[,2])

nF = 480

#Seriesplot.fn(Data[1:nF,1]-2000, Data[1:nF,2], points=FALSE,

# xlab="Time in Seconds", ylab="Air Flow")

plot( Data[1:nF,1]-2000, Data[1:nF,2], axes=FALSE, type="n",

xlab="Time in Seconds",ylab="Air Flow",

main="A", cex.lab=1.6, cex.main=2)

axis(1); axis(2, las=2); box()

#points(Data[1:nF,1]-2000, Data[1:nF,2], cex=0.5)

lines(Data[1:nF,1]-2000, Data[1:nF,2], cex=2)

lines(Data[1:nF,1]-2000, fit.MA[1:nF], col=2)

lines(Data[1:nF,1]-2000, fit.LPF[1:nF], col=3)

legend( "bottomright", legend=c("Raw data", "Moving average", "Low pass filter"),

col=1:3, lty=rep(1,3) )

##### Figure 1B: IBI data

Fs=50 ## sampling frequency is 50Hz

Peaks <- find.peaks(Data[,2], Fs, lowpass=TRUE, freq=1, MovingAv=FALSE,

W=FALSE, filter=TRUE, threshold=0.05)

#points(Data[Peaks[2:13,1],1],Data[Peaks[2:13,1],2],col=2)

PP_interval <- diff(Peaks[,1])/Fs

plot( 1:length(PP_interval), PP_interval, axes=FALSE, type="n",

xlab="Air flow cycle series", ylab="IBI in seconds",

main="B", cex.lab=1.6, cex.main=2)

axis(1); axis(2, las=2); box()

lines( 1:length(PP_interval), PP_interval)

par( mfrow=c(1,2), mar=c(5.1, 5.1, 3.1, 2.1) )

##### Figure 1C: MSE

scale_raw <- seq(1,33) #scale_raw <- seq(1, 90, 2)

scale_PP <- scale_raw

mse.raw <- MSE(Data$V2[seq(1,100000,2)], tau=scale_raw, m=2, r=0.15, I=40000)

mse.IBI <- MSE(PP_interval, tau=scale_PP, m=2, r=0.15, I=40000)

plot( mse.raw$tau ,mse.raw$SampEn, axes=FALSE, xlab="Scale",ylab="Sample entropy",

main="C", cex.lab=1.6)#, cex.main=1)

axis(1, cex=2); axis(2, las=2); box()

lines(mse.raw$tau ,mse.raw$SampEn)

points(mse.IBI$tau ,mse.IBI$SampEn, col=2)

lines(mse.IBI$tau ,mse.IBI$SampEn, col=2)

legend( "bottomright", legend=c("Raw data of air flow", "IBI of air flow"),

col=1:2, lty=1:2, pch=rep(1,1) )

#### Figure 1D: PSD analysis

LowPSD(PP_interval, plot=TRUE,min=1/64, max=1/2)

title(main="D")

########################### Figure 2 ############################

#par( mfrow=c(3,2), mar=c(5.1, 5.1, 3.1, 2.1) )

#par(mfrow=c(3,2))

#### MFDFA: Figure 2A-D

exponents=seq(3, 9, by=1/4)

scale=2^exponents

q=-10:10

m=2

Result <- MFDFA(PP_interval, scale, m, q)

MFDFAplot.fn(Result, scale, q, model = TRUE)

#### fit.model

Coeff <- fit.model(Result$Hq,q)

Coeff

# a b Goodness

#0.4538859 0.7752902 0.9965735

Para<- -log(Coeff)/log(2);Para[3]=Para[1]-Para[2]

names(Para)<-c("Hmax","Hmin","deltaH")

Para

# Hmax Hmin deltaH

#1.1395984 0.3671916 0.7724067

par(mfrow=c(1,2))

#### Individualplot

data("HqData")

PP_Hq <- HqData

filenames <- row.names(PP_Hq)

q=-10:10

ClassNames <- c(substr(filenames[1:19], start = 1, stop = 3),

substr(filenames[20:38], start = 1, stop = 5))

Class <- unique(ClassNames)

col_vec <- rep(NA, nrow(PP_Hq) )

pch_vec <- rep(16, nrow(PP_Hq) )

for( i in 1:length(Class) ) { col_vec[ ClassNames == Class[i] ] <- i }

Individualplot.fn(q,PP_Hq,Name=Class,col=col_vec,pch=pch_vec, xlab="q",ylab="Hurst exponent")

legend("topright", legend=paste0(Class, "(N=", table( ClassNames ), ")"),

col=1:4, cex=1, lty=1, pch=16, bg = "white")

title(main="E: individuals")

#### Groupplot

data("HqData")

PP_Hq <- HqData

filenames <- row.names(PP_Hq)

q <- -10:10

ClassNames <- c(substr(filenames[1:19], start = 1, stop = 3),

substr(filenames[20:38], start = 1, stop = 5))

Class <- unique(ClassNames)

for (i in 1:length(q)){

Data <- GroupComparison.fn(PP_Hq[,i],ClassNames)

Result_mean_vec <- Data[,"Mean"]

Result_sd_vec <- Data[,"SE"]

if( i == 1 ) {

Result_mean_mat <- Result_mean_vec

Result_sd_mat <- Result_sd_vec

} else {

Result_mean_mat <- rbind(Result_mean_mat, Result_mean_vec)

Result_sd_mat <- rbind(Result_sd_mat, Result_sd_vec)

}

}

Groupplot.fn(q, Result_mean_mat, Class, errorbar = Result_sd_mat,

xRange = NA, yRange = NA, col = NA, pch = rep(16,4), Position = "topright",

cex.legend = 1, xlab="q",ylab="Hurst exponent",main = "")

title(main="F: Groups")

**References**

[1] Costa M, Goldberger AL, Peng CK. Multiscale entropy analysis of complex physiologic time series. Phys Rev Lett 2002;89:068102.

[2] Eke A, Hermán P, Bassingthwaighte JB, Raymond GM, Percival DB, Cannon M, et al. Physiological time series: distinguishing fractal noises from motions. Pflugers Arch 2000;439:403-15.

[3] Kantelhardt JW, Zschiegner SA, Koscielny-Bunde E, Havlin S, Bunde A, Stanley HE. Multifractal detrended fluctuation analysis of nonstationary time series. Physica A: Statistical Mechanics and its Applications 2002;316:87-114.

[4] Kantelhardt JW. Fractal and Multifractal Time Series. In: Meyers R. A. (ed) Encyclopedia of Complexity and Systems Science. New York, NY: Springer New York, 2009, 3754-79.

[5] Gu GF, Zhou WX. Detrended fluctuation analysis for fractals and multifractals in higher dimensions. Phys Rev E Stat Nonlin Soft Matter Phys 2006;74:061104.

[6] Zhou Y, Leung Y, Yu ZG. Relationships of exponents in two-dimensional multifractal detrended fluctuation analysis. Phys Rev E Stat Nonlin Soft Matter Phys 2013;87:012921.

[7] Macek WM. Multifractality and intermittency in the solar wind. Nonlin. Processes Geophys. 2007;14:695-700.
